# 4-Methyl-*N*-(3-methyl­phen­yl)benzene­sulfonamide

**DOI:** 10.1107/S1600536809049332

**Published:** 2009-11-25

**Authors:** P. G. Nirmala, B. Thimme Gowda, Sabine Foro, Hartmut Fuess

**Affiliations:** a Department of Chemistry, Mangalore University, Mangalagangotri 574 199, Mangalore, India; b Institute of Materials Science, Darmstadt University of Technology, Petersenstrasse 23, D-64287 Darmstadt, Germany

## Abstract

In the title compound, C_14_H_15_NO_2_S, the conformation of the N—C bond in the C—SO_2_—NH—C segment has *gauche* torsion angles with respect to the S=O bonds. Further, the conformation of the N—H bond is *anti* to the 3-methyl group in the aniline benzene ring. The mol­ecule is bent at the N atom with a C—SO_2_—NH—C torsion angle of 56.7 (3)°. The dihedral angle between the benzene rings is 83.9 (1)°. In the crystal, inter­molecular N—H⋯O hydrogen bonds pack the mol­ecules into a supra­molecular structure.

## Related literature

For the preparation of the title compound, see: Gowda *et al.* (2005 ▶). For a study of the effect of substituents on the crystal structures of *N*-(ar­yl)-aryl­sulfonamides, see: Gowda *et al.* (2009**a* ▶,b* ▶); Nirmala *et al.*(2009 ▶). For bond lengths in other aryl sulfonamides, see: Gelbrich *et al.* (2007 ▶); Perlovich *et al.* (2006 ▶).
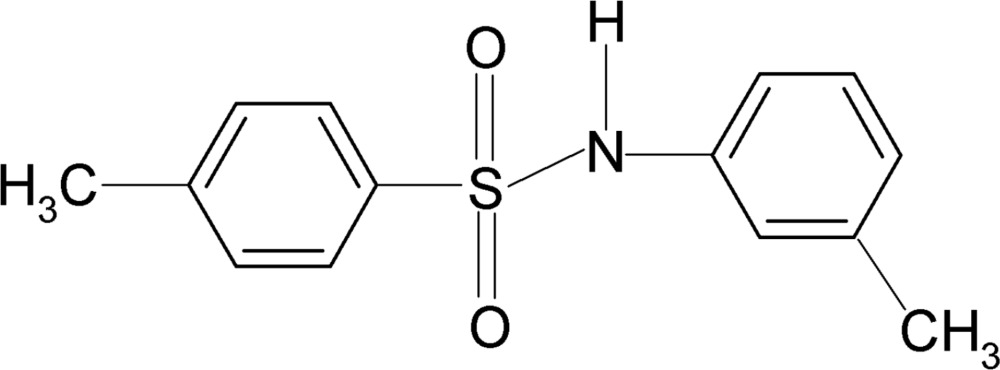


## Experimental

### 

#### Crystal data

C_14_H_15_NO_2_S
*M* *_r_* = 261.33
Monoclinic, 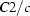

*a* = 14.076 (3) Å
*b* = 14.519 (3) Å
*c* = 13.482 (2) Å
β = 98.10 (2)°
*V* = 2727.8 (9) Å^3^
*Z* = 8
Cu *K*α radiation
μ = 2.06 mm^−1^
*T* = 299 K
0.50 × 0.45 × 0.35 mm

#### Data collection

Enraf–Nonius CAD-4 diffractometer
Absorption correction: ψ scan (North *et al.*, 1968 ▶) *T*_min_ = 0.426, *T*_max_ = 0.533
5405 measured reflections
2436 independent reflections
2285 reflections with *I* > 2σ(*I*)
*R*_int_ = 0.148
3 standard reflections frequency: 120 min intensity decay: 1.5%

#### Refinement

*R*[*F* ^2^ > 2σ(*F* ^2^)] = 0.061
*wR*(*F* ^2^) = 0.163
*S* = 1.05
2436 reflections
169 parameters
13 restraints
H atoms treated by a mixture of independent and constrained refinement
Δρ_max_ = 0.42 e Å^−3^
Δρ_min_ = −0.42 e Å^−3^

### 

Data collection: *CAD-4-PC* (Enraf–Nonius, 1996 ▶); cell refinement: *CAD-4-PC*; data reduction: *REDU4* (Stoe & Cie, 1987 ▶); program(s) used to solve structure: *SHELXS97* (Sheldrick, 2008 ▶); program(s) used to refine structure: *SHELXL97* (Sheldrick, 2008 ▶); molecular graphics: *PLATON* (Spek, 2009 ▶); software used to prepare material for publication: *SHELXL97*.

## Supplementary Material

Crystal structure: contains datablocks I, global. DOI: 10.1107/S1600536809049332/lx2127sup1.cif

Structure factors: contains datablocks I. DOI: 10.1107/S1600536809049332/lx2127Isup2.hkl

Additional supplementary materials:  crystallographic information; 3D view; checkCIF report

## Figures and Tables

**Table 1:** Hydrogen-bond geometry (Å, °)

*D*—H⋯*A*	*D*—H	H⋯*A*	*D*⋯*A*	*D*—H⋯*A*
N1—H1*N*⋯O2^i^	0.839 (18)	2.10 (2)	2.914 (3)	163 (3)

Symmetry code: (i) 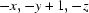.

**Fig. 1.**
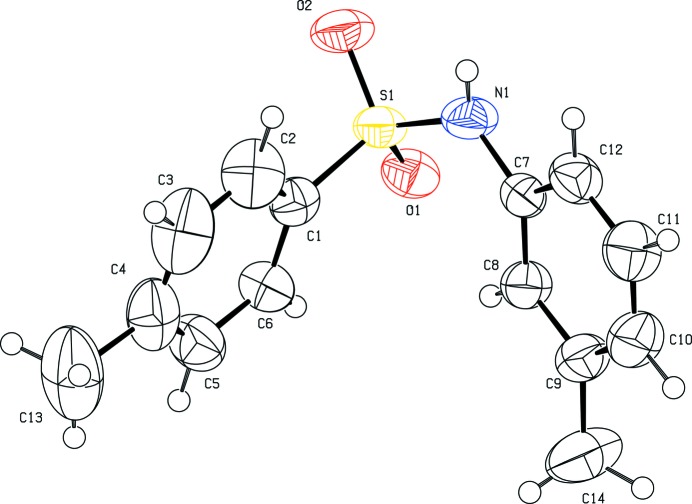
Molecular structure of (I), showing the atom labelling scheme and displacement ellipsoids drawn at the 50% probability level.

**Fig. 2.**
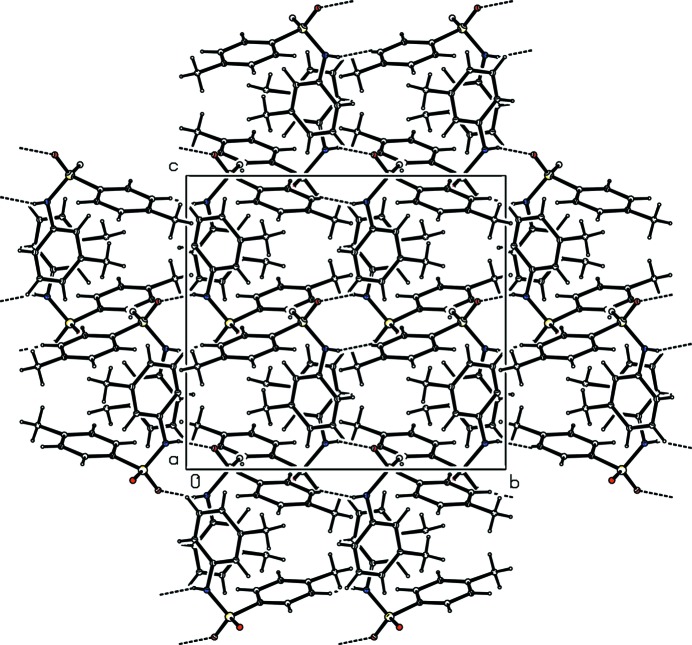
Molecular packing of (I) with hydrogen bonding shown as dashed lines.

## supplementary crystallographic information

### Comment

As part of a study of the effect of substituents on the crystal structures of *N*–(aryl)–arylsulfonamides (Gowda *et al.*, 2009*a,b*; Nirmala *et al.*, 2009), in the present work, the structure of 4–methyl–*N*–(3–methylphenyl)benzenesulfonamide (I) has been determined.

The conformation of the N—C bond in the C1—SO_2_—NH—C7 segment of the structure has *gauche* torsions with respect to the SO bonds (Fig. 1). Further, the conformation of the N—H bond is *anti* to the 3–methyl group in the aniline benzene ring. The molecule is bent at the *N* atom with the C1—SO_2_—NH—C7 torsion angle of 56.7 (3)°, compared to the values of -51.6 (3)° in 4–methyl–*N*–(phenyl)–benzenesulfonamide (II)(Gowda *et al.*, 2009*b*), 60.0 (2)° in 4–methyl–*N*–(2–methylphenyl)benzenesulfonamide (III) (Nirmala *et al.*, 2009) and -61.8 (2)° in 4–methyl–*N*–(3,4–dimethylphenyl)benzenesulfonamide (IV) (Gowda *et al.*, 2009*a*). The two benzene rings in (I) are tilted relative to each other by 83.9 (1)°, compared to the values of 68.4 (1)° in (II), 49.7 (1)° in (III) and 47.8 (1)° in (IV). The other bond parameters are similar to those observed in (II), (III), (IV) and other aryl sulfonamides (Perlovich *et al.*, 2006; Gelbrich *et al.*, 2007). The crystal packing stabilized by intermolecular N—H···O hydrogen bonds (Table 1) is shown in Fig.2.

### Experimental

The solution of toluene (10 ml) in chloroform (40 ml) was treated dropwise with chlorosulfonic acid (25 ml) at 0 ° C. After the initial evolution of hydrogen chloride subsided, the reaction mixture was brought to room temperature and poured into crushed ice in a beaker. The chloroform layer was separated, washed with cold water and allowed to evaporate slowly. The residual benzenesulfonylchloride was treated with *m*–toluidine in the stoichiometric ratio and boiled for ten minutes. The reaction mixture was then cooled to room temperature and added to ice cold water (100 ml). The resultant solid 4–methyl–*N*–(3–methylphenyl)benzenesulfonamide was filtered under suction and washed thoroughly with cold water. It was then recrystallized to constant melting point from dilute ethanol. The purity of the compound was checked and characterized by recording its infrared and NMR spectra (Gowda *et al.*, 2005). The single crystals used in X-ray diffraction studies were grown in ethanolic solution by a slow evaporation at room temperature.

### Refinement

The H atom of the NH group was located in a difference map and later restrained to the distance N—H = 0.84 (2) Å. The other H atoms were positioned with idealized geometry using a riding model [C—H = 0.93–0.96 Å]. All H atoms were refined with isotropic displacement parameters (set to 1.2 times of the *U*_eq_ of the parent atom). The U^ij^ components of C3 and C4 were restrained to approximate isotropic behavoir.

### Crystal data

**Table:**

C_14_H_15_NO_2_S	*F*(000) = 1104
*M**_r_* = 261.33	*D*_x_ = 1.273 Mg m^−^^3^
Monoclinic, *C*2/*c*	Cu *K*α radiation, λ = 1.54180 Å
Hall symbol: -C 2yc	Cell parameters from 25 reflections
*a* = 14.076 (3) Å	θ = 5.2–23.5°
*b* = 14.519 (3) Å	µ = 2.06 mm^−^^1^
*c* = 13.482 (2) Å	*T* = 299 K
β = 98.10 (2)°	Prism, colourless
*V* = 2727.8 (9) Å^3^	0.50 × 0.45 × 0.35 mm
*Z* = 8	

### Data collection

**Table:**

Enraf–Nonius CAD-4 diffractometer	2285 reflections with *I* > 2σ(*I*)
Radiation source: fine-focus sealed tube	*R*_int_ = 0.148
graphite	θ_max_ = 67.0°, θ_min_ = 4.4°
ω/2θ scans	*h* = −16→12
Absorption correction: ψ scan (North *et al.*, 1968)	*k* = −12→17
*T*_min_ = 0.426, *T*_max_ = 0.533	*l* = −16→16
5405 measured reflections	3 standard reflections every 120 min
2436 independent reflections	intensity decay: 1.5%

### Refinement

**Table:**

Refinement on *F*^2^	Secondary atom site location: difference Fourier map
Least-squares matrix: full	Hydrogen site location: inferred from neighbouring sites
*R*[*F*^2^ > 2σ(*F*^2^)] = 0.061	H atoms treated by a mixture of independent and constrained refinement
*wR*(*F*^2^) = 0.163	*w* = 1/[σ^2^(*F*_o_^2^) + (0.0638*P*)^2^ + 2.2735*P*] where *P* = (*F*_o_^2^ + 2*F*_c_^2^)/3
*S* = 1.05	(Δ/σ)_max_ < 0.001
2436 reflections	Δρ_max_ = 0.42 e Å^−^^3^
169 parameters	Δρ_min_ = −0.42 e Å^−^^3^
13 restraints	Extinction correction: *SHELXL97* (Sheldrick, 2008), Fc^*^=kFc[1+0.001xFc^2^λ^3^/sin(2θ)]^-1/4^
Primary atom site location: structure-invariant direct methods	Extinction coefficient: 0.0092 (7)

### Special details

**Table:**

Geometry. All e.s.d.'s (except the e.s.d. in the dihedral angle between two l.s. planes) are estimated using the full covariance matrix. The cell e.s.d.'s are taken into account individually in the estimation of e.s.d.'s in distances, angles and torsion angles; correlations between e.s.d.'s in cell parameters are only used when they are defined by crystal symmetry. An approximate (isotropic) treatment of cell e.s.d.'s is used for estimating e.s.d.'s involving l.s. planes.
Refinement. Refinement of *F*^2^ against ALL reflections. The weighted *R*-factor *wR* and goodness of fit *S* are based on *F*^2^, conventional *R*-factors *R* are based on *F*, with *F* set to zero for negative *F*^2^. The threshold expression of *F*^2^ > σ(*F*^2^) is used only for calculating *R*-factors(gt) *etc*. and is not relevant to the choice of reflections for refinement. *R*-factors based on *F*^2^ are statistically about twice as large as those based on *F*, and *R*- factors based on ALL data will be even larger.

### Fractional atomic coordinates and isotropic or equivalent isotropic displacement parameters (Å^2^)

**Table:**

	*x*	*y*	*z*	*U*_iso_*/*U*_eq_	
C1	0.03543 (17)	0.26890 (18)	0.04684 (16)	0.0478 (6)	
C2	−0.0487 (2)	0.2802 (3)	0.0874 (3)	0.0769 (9)	
H2	−0.0754	0.3384	0.0915	0.092*	
C3	−0.0921 (2)	0.2041 (4)	0.1217 (3)	0.0927 (13)	
H3	−0.1487	0.2117	0.1490	0.111*	
C4	−0.0551 (3)	0.1180 (3)	0.1169 (2)	0.0767 (10)	
C5	0.0301 (2)	0.1079 (3)	0.0788 (2)	0.0706 (8)	
H5	0.0576	0.0499	0.0768	0.085*	
C6	0.0746 (2)	0.1826 (2)	0.0439 (2)	0.0576 (7)	
H6	0.1319	0.1748	0.0180	0.069*	
C7	0.17516 (16)	0.41609 (16)	0.18078 (17)	0.0450 (5)	
C8	0.24019 (18)	0.34436 (19)	0.19055 (18)	0.0509 (6)	
H8	0.2450	0.3058	0.1364	0.061*	
C9	0.2985 (2)	0.3297 (2)	0.2811 (2)	0.0584 (7)	
C10	0.2903 (2)	0.3882 (3)	0.3600 (2)	0.0690 (8)	
H10	0.3293	0.3793	0.4208	0.083*	
C11	0.2259 (2)	0.4588 (2)	0.3501 (2)	0.0695 (8)	
H11	0.2217	0.4975	0.4043	0.083*	
C12	0.1671 (2)	0.47364 (19)	0.2610 (2)	0.0561 (6)	
H12	0.1227	0.5215	0.2548	0.067*	
C13	−0.1073 (4)	0.0352 (4)	0.1498 (3)	0.1229 (19)	
H13A	−0.1598	0.0199	0.0990	0.148*	
H13B	−0.1314	0.0491	0.2112	0.148*	
H13C	−0.0639	−0.0160	0.1601	0.148*	
C14	0.3672 (3)	0.2506 (3)	0.2921 (3)	0.0958 (13)	
H14A	0.3922	0.2412	0.2302	0.115*	
H14B	0.3346	0.1959	0.3088	0.115*	
H14C	0.4191	0.2638	0.3444	0.115*	
N1	0.11662 (17)	0.43561 (15)	0.08911 (16)	0.0578 (6)	
H1N	0.0746 (19)	0.475 (2)	0.096 (3)	0.069*	
O1	0.17370 (14)	0.33270 (13)	−0.03761 (13)	0.0573 (5)	
O2	0.01925 (16)	0.41182 (14)	−0.07129 (14)	0.0680 (6)	
S1	0.08931 (4)	0.36406 (4)	−0.00253 (4)	0.0483 (3)	

### Atomic displacement parameters (Å^2^)

**Table:**

	*U*^11^	*U*^22^	*U*^33^	*U*^12^	*U*^13^	*U*^23^
C1	0.0524 (12)	0.0520 (14)	0.0383 (11)	0.0040 (11)	0.0037 (9)	−0.0048 (10)
C2	0.0713 (18)	0.087 (2)	0.0776 (19)	0.0045 (18)	0.0286 (15)	−0.0209 (18)
C3	0.0718 (19)	0.137 (4)	0.076 (2)	−0.022 (2)	0.0362 (16)	−0.023 (2)
C4	0.088 (2)	0.095 (2)	0.0472 (14)	−0.033 (2)	0.0092 (14)	−0.0032 (15)
C5	0.0826 (19)	0.0594 (17)	0.0694 (18)	−0.0097 (17)	0.0089 (15)	0.0091 (15)
C6	0.0628 (14)	0.0497 (14)	0.0613 (15)	0.0025 (13)	0.0125 (11)	0.0044 (12)
C7	0.0516 (12)	0.0368 (11)	0.0452 (12)	−0.0016 (10)	0.0020 (9)	0.0014 (9)
C8	0.0592 (14)	0.0476 (13)	0.0442 (12)	0.0045 (12)	0.0014 (10)	−0.0033 (10)
C9	0.0624 (14)	0.0568 (16)	0.0521 (14)	0.0069 (13)	−0.0054 (11)	0.0012 (12)
C10	0.0826 (19)	0.0710 (19)	0.0478 (14)	0.0012 (17)	−0.0098 (13)	−0.0047 (14)
C11	0.0908 (19)	0.0649 (18)	0.0508 (14)	0.0009 (17)	0.0033 (13)	−0.0156 (13)
C12	0.0669 (14)	0.0449 (14)	0.0563 (14)	0.0021 (12)	0.0078 (11)	−0.0050 (11)
C13	0.141 (4)	0.152 (5)	0.078 (2)	−0.078 (4)	0.023 (2)	0.011 (3)
C14	0.102 (3)	0.100 (3)	0.076 (2)	0.042 (2)	−0.0205 (19)	−0.007 (2)
N1	0.0746 (14)	0.0409 (11)	0.0528 (12)	0.0174 (11)	−0.0088 (10)	−0.0052 (9)
O1	0.0713 (11)	0.0513 (10)	0.0504 (9)	0.0047 (9)	0.0127 (8)	0.0068 (8)
O2	0.0909 (14)	0.0553 (11)	0.0502 (10)	0.0221 (11)	−0.0162 (9)	0.0009 (8)
S1	0.0622 (5)	0.0406 (4)	0.0400 (4)	0.0101 (2)	0.0004 (3)	0.0017 (2)

### Geometric parameters (Å, °)

**Table:**

C1—C6	1.372 (4)	C9—C10	1.378 (4)
C1—C2	1.382 (4)	C9—C14	1.496 (5)
C1—S1	1.752 (3)	C10—C11	1.362 (5)
C2—C3	1.375 (6)	C10—H10	0.9300
C2—H2	0.9300	C11—C12	1.377 (4)
C3—C4	1.358 (7)	C11—H11	0.9300
C3—H3	0.9300	C12—H12	0.9300
C4—C5	1.378 (5)	C13—H13A	0.9600
C4—C13	1.508 (5)	C13—H13B	0.9600
C5—C6	1.369 (4)	C13—H13C	0.9600
C5—H5	0.9300	C14—H14A	0.9600
C6—H6	0.9300	C14—H14B	0.9600
C7—C8	1.380 (4)	C14—H14C	0.9600
C7—C12	1.384 (4)	N1—S1	1.619 (2)
C7—N1	1.414 (3)	N1—H1N	0.839 (18)
C8—C9	1.388 (3)	O1—S1	1.414 (2)
C8—H8	0.9300	O2—S1	1.4335 (18)
			
C6—C1—C2	119.4 (3)	C9—C10—H10	119.5
C6—C1—S1	120.79 (19)	C10—C11—C12	120.8 (3)
C2—C1—S1	119.8 (2)	C10—C11—H11	119.6
C3—C2—C1	119.0 (3)	C12—C11—H11	119.6
C3—C2—H2	120.5	C11—C12—C7	118.8 (3)
C1—C2—H2	120.5	C11—C12—H12	120.6
C4—C3—C2	122.1 (3)	C7—C12—H12	120.6
C4—C3—H3	118.9	C4—C13—H13A	109.5
C2—C3—H3	118.9	C4—C13—H13B	109.5
C3—C4—C5	118.4 (4)	H13A—C13—H13B	109.5
C3—C4—C13	120.9 (4)	C4—C13—H13C	109.5
C5—C4—C13	120.7 (4)	H13A—C13—H13C	109.5
C6—C5—C4	120.6 (4)	H13B—C13—H13C	109.5
C6—C5—H5	119.7	C9—C14—H14A	109.5
C4—C5—H5	119.7	C9—C14—H14B	109.5
C5—C6—C1	120.5 (3)	H14A—C14—H14B	109.5
C5—C6—H6	119.7	C9—C14—H14C	109.5
C1—C6—H6	119.7	H14A—C14—H14C	109.5
C8—C7—C12	120.5 (2)	H14B—C14—H14C	109.5
C8—C7—N1	122.1 (2)	C7—N1—S1	125.80 (17)
C12—C7—N1	117.4 (2)	C7—N1—H1N	112 (2)
C7—C8—C9	120.1 (2)	S1—N1—H1N	115 (2)
C7—C8—H8	119.9	O1—S1—O2	118.24 (12)
C9—C8—H8	119.9	O1—S1—N1	109.97 (12)
C10—C9—C8	118.7 (3)	O2—S1—N1	104.53 (12)
C10—C9—C14	121.4 (3)	O1—S1—C1	107.58 (11)
C8—C9—C14	119.9 (3)	O2—S1—C1	109.39 (13)
C11—C10—C9	121.1 (3)	N1—S1—C1	106.57 (12)
C11—C10—H10	119.5		
			
C6—C1—C2—C3	−1.5 (4)	C9—C10—C11—C12	0.1 (5)
S1—C1—C2—C3	177.1 (2)	C10—C11—C12—C7	−0.8 (5)
C1—C2—C3—C4	0.0 (5)	C8—C7—C12—C11	0.9 (4)
C2—C3—C4—C5	1.8 (5)	N1—C7—C12—C11	−177.3 (3)
C2—C3—C4—C13	−176.6 (4)	C8—C7—N1—S1	21.5 (4)
C3—C4—C5—C6	−1.9 (5)	C12—C7—N1—S1	−160.3 (2)
C13—C4—C5—C6	176.4 (3)	C7—N1—S1—O1	−59.6 (3)
C4—C5—C6—C1	0.4 (5)	C7—N1—S1—O2	172.5 (2)
C2—C1—C6—C5	1.4 (4)	C7—N1—S1—C1	56.7 (3)
S1—C1—C6—C5	−177.3 (2)	C6—C1—S1—O1	−1.9 (2)
C12—C7—C8—C9	−0.3 (4)	C2—C1—S1—O1	179.5 (2)
N1—C7—C8—C9	177.8 (3)	C6—C1—S1—O2	127.7 (2)
C7—C8—C9—C10	−0.4 (4)	C2—C1—S1—O2	−50.9 (3)
C7—C8—C9—C14	178.6 (3)	C6—C1—S1—N1	−119.8 (2)
C8—C9—C10—C11	0.5 (5)	C2—C1—S1—N1	61.6 (2)
C14—C9—C10—C11	−178.4 (4)		

### Hydrogen-bond geometry (Å, °)

**Table:**

*D*—H···*A*	*D*—H	H···*A*	*D*···*A*	*D*—H···*A*
N1—H1N···O2^i^	0.84 (2)	2.10 (2)	2.914 (3)	163 (3)

Symmetry codes: (i) −*x*, −*y*+1, −*z*.
